# Cardioprotective Effect of Tangeretin by Inhibiting PTEN/AKT/mTOR Axis in Experimental Sepsis-Induced Myocardial Dysfunction

**DOI:** 10.3390/molecules25235622

**Published:** 2020-11-29

**Authors:** Predeepkumar Narayanappa Shiroorkar, Obaid Afzal, Imran Kazmi, Fahad A. Al-Abbasi, Abdulmalik Saleh Alfawaz Altamimi, Kumar Shiva Gubbiyappa, Nagaraja Sreeharsha

**Affiliations:** 1 Department of Basic Medical Sciences, College of Medicine, King Faisal University, Al-Ahsa 31982, Saudi Arabia; 2 Department of Pharmaceutical Chemistry, College of Pharmacy, Prince Sattam Bin Abdulaziz University, AlKharj 11942, Saudi Arabia; o.akram@psau.edu.sa (O.A.); as.altamimi@psau.edu.sa (A.S.A.A.); 3 Department of Biochemistry, Faculty of Science, King Abdulaziz University, Jeddah 21589, Saudi Arabia; kazmiimran2005@gmail.com (I.K.); fabbasi@kau.edu.sa (F.A.A.-A.); 4 School of Pharmacy, School of Pharmacy, GITAM Deemed to be University, Hyderabad 502329, India; sgubbiya@gitam.edu; 5 Department of Pharmaceutical Sciences, College of Clinical Pharmacy, King Faisal University, Al-Ahsa 31982, Saudi Arabia; 6 Department of Pharmaceutics, Vidya Siri College of Pharmacy, Off Sarjapura Road, Bangalore 560035, India

**Keywords:** cecum ligation, puncture, tangeretin, sepsis, PTEN, AKT, mTOR

## Abstract

Sepsis aggregates undesirable immune response causing depression of ventricular myocardium and diastolic dysfunction. This present study examined the effect of a plant-derived flavone tangeretin (TG) on autophagy and reduction in myocardial dysfunction. The sepsis was induced by cecum ligation and puncture (CLP) in male Sprague–Dawley rats. Abnormal changes were seen in the heart after the sepsis induction. These abnormalities were analyzed based on the cardiac markers, namely Cardiac myosin light chain-1 (cMLC1) and Cardiac troponin I (cTnl), echocardiography, and plasma parameters, like Lactate dehydrogenase (LDH) and Creatinine kinase (CK). Microanatomy of the heart was studied using hematoxylin and eosin stained histopathological samples of cardiac tissue. Western blot technique was used to detect the nature and extent of protein with the amount of a specific RNA (gene expression) in the cardiac homogenate. Oxidative damage was analyzed using redox marker, reduced glutathione. This study successfully showed that TG attenuated sepsis-induced myocardial dysfunction by inhibiting myocardial autophagy via silencing the Phosphatase and tensin homolog (PTEN) expression and acting on the AKT/mTOR pathway. The present findings supported that TG is a novel cardioprotective therapeutic target for sepsis induced myocardial dysfunction.

## 1. Introduction

Sepsis is one of the most acute and critical disease complications resulting in systemic inflammatory response syndrome and is usually caused by various microorganisms, such as bacteria, fungi, etc. This condition leads to multiple organ damage and/or failure and typically it leads to endothelial dysfunction, acute lung injury, suppression of bone marrow, disorder in acid-based balance, liver injury, dysfunction of the blood coagulation, and myocardial damage. Statistics indicate that myocardial injuries are exhibited in 40% to 50% of sepsis patients, and studies performed till now are not able to explain the mechanism of sepsis leading to cardiac injury [1,2]. For the removal of damaged organelles, including mitochondria from the cellular milieu, there is a bulk regulated pathway that is highly regulated and known as autophagy. Autophagy preserves cellular homeostasis and survival. Though adverse effects may be exerted on the cellular health and cardiac function due to excessive autophagy, and, therefore, key to reducing septic myocardial damage is a moderate degree of autophagy. Tangeretin (TG), a flavonoid has widespread pharmacological activities, including anti-inflammatory, antitumor, anti-asthmatic, and antioxidative. Studies have shown that TG has protective action against several acute damages [3,4,5,6,7]. 

Our current study emphasizes investigating the role of TG in cecum ligation and perforation (CLP) induced septic myocardial autophagy in rats and understanding its mechanism of cardio-protection.

## 2. Results

### 2.1. CLP Induced Sepsis and Sepsis Mediated Myocardial Dysfunction

CLP was performed in rats for the analyzing the cardioprotective effect TG on sepsis-induced myocardial damage. The effect was analyzed at 2 dose levels, namely 50 and 100 mg/kg.

**Figure 1:**
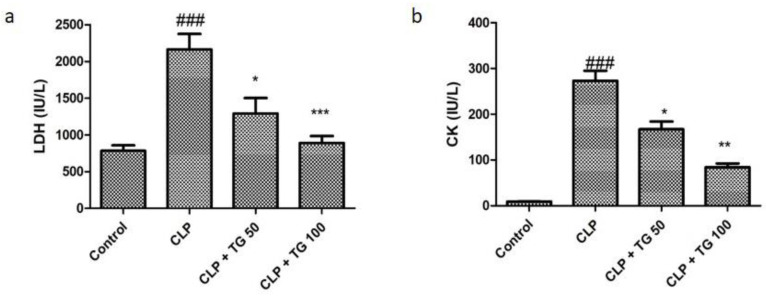
Cardiac injuries were measured by plasma. (**a**) Lactate dehydrogenase (LDH) and (**b**) Creatinine kinase (CK). LDH and CK plasma levels were increased in cecum ligation and puncture (CLP)-treated rats. The CLP-induced cardiac injury was significantly attenuated by tangeretin (TG) (50 and 100 mg/kg) treatment, dose dependently. One-way analysis of variance (ANOVA), followed by Tukey’s test were used for statically analysis (Significance level * *p* < 0.05). Values represented as means ± SD and n = 12/group. Where, ### *p* < 0.001 when compared to control group, whereas * *p* < 0.05, ** *p* < 0.01 and *** *p* < 0.001 when compared CLP group animals.

Figure 1 shows the increased levels of lactate dehydrogenase (LDH, Figure 1a), and Creatinine kinase (CK, Figure 1b) in diseased animals (Control group), which manifests the CLP-induced myocardial injury. This injury was significantly decreased with treatment of TG and in dose-dependent manner.

### 2.2. TUNEL Staining, Cell Death Markers, DNA Fragmentation, and PARP Activity

**Figure 2:**
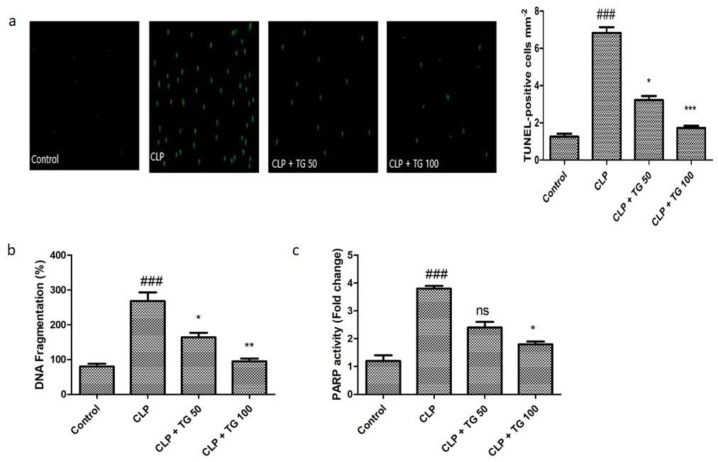
(**a**) Representative fluorescent images of TUNEL staining performed on paraffin section of rat heart in each group; Scale bar 5 mm= 25 µm. Green color demonstrated TUNEL-positive nuclei and intensity of the same is plotted as bar graph near the TUNEL images. (**b**) Cardiac cell death markers DNA fragmentation and (**c**) poly(ADP-ribose) polymerase (PARP) activity assay. Both DNA fragmentation and PARP activity were significantly increased in CLP-rats, and TG (50 and 100 mg/kg) treatment ameliorated elevated levels, dose-dependently. Values represented as means ± SD and n = 12/group. One-way analysis of variance (ANOVA), followed by Tukey’s test were used for statically analysis (Significance level * *p* < 0.05). Where ### *p* < 0.001 compared to control group, whereas ns, * *p* < 0.05, ** *p* < 0.01 and *** *p* < 0.001 compared with CLP group animals.

Different cell death was observed by TUNEL (Terminal deoxynucleotidyl transferase (TdT) biotin-dUTP nick end labeling) staining technique (Figure 2a). It helps to determine the programmed cell death or apoptosis. Treating tissue sections with TdT and labeled nucleotides provides a convenient assay for apoptosis. The nuclear enzyme poly(ADP-ribose) polymerase (PARP) is a key enzyme indicative of cell death or survival in cardiovascular diseases. Oxidative stress induces DNA damage that activates PARP enzyme, leading to poly (ADP-ribosyl) ation of nuclear proteins. PARP activity and quantitative cell death were increased in response to CLP induced myocardial injury by 398%. Separation of deoxyribonucleic acid strands into pieces was found to decrease by 55% and 57% in the TG treatment groups dosed 50 and 100 mg/kg, respectively (Figure 2b). PARP activity was also reduced significantly after treatment with TG in dose-dependent manner (Figure 2c).

### 2.3. Analysis of Acute Myocardial Injury

**Figure 3:**
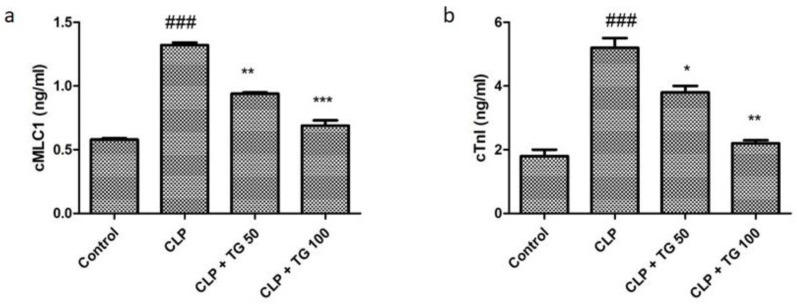
Damage was measured by plasma (**a**) cTnI and (**b**) Cardiac myosin light chain-1 (cMLC1), which were secreted by damaged cardiomyocytes from the heart. Both of them were significantly increased in CLP-rats and were significantly attenuated by TG (50 and 100 mg/kg) treatment, dose-dependently. Values represented as means ± SD; n = 12/group. One-way analysis of variance (ANOVA), followed by Tukey’s test were used for statically analysis (Significance level * *p* < 0.05). Where, ### *p* < 0.001 compared to control group, whereas * *p* < 0.05, ** *p* < 0.01 and *** *p* < 0.001 compared CLP group of animals.

Two potential biomarkers of the acute myocardial injury are Cardiac myosin light chain-1 (cMLC1) and Cardiac troponin I (cTnl). Normal rats showed cMLC1 and cTnl concentrations in serum 0.62 ng/mL and 1.8 ng/mL, respectively. CLP induced sepsis mediated myocardial injury significantly elevated these biomarkers in serum. Treatment with TG 50 and 100 mg/kg significantly reduced the serum cMLC1 to 0.82 and 0.73 ng/mL, respectively (Figure 3a). Treatment with TG 50 and 100 mg/kg significantly reduced CLP-induced serum cTnI levels, as well to 3.8 and 2.1, respectively (Figure 3b).

### 2.4. Echocardiography for Cardiac Functioning

**Figure 4:**
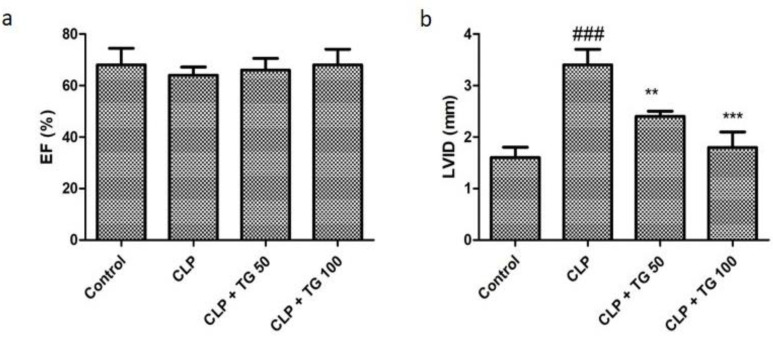
Cardiac function parameters. (**a**) Ejection fraction (EF) and (**b**) Left ventricular internal dimension (LVID) were measured by echocardiography. LVID was significantly increased whereas effect on EF was non-significant in CLP rats. TG (50 and 100 mg/kg) reversed the change in LVID in dose dependent manner. Values represented as means ± SD and n = 12/group. One-way analysis of variance (ANOVA), followed by Tukey’s test were used for statically analysis. Where ### *p* < 0.001 compared to control group, whereas ** *p* < 0.01 and *** *p* < 0.001 when compared with CLP group animals.

The anatomy and physiology of left ventricles of heart was observed by echocardiography. CLP did not affect the ejection fraction (EF) of the heart (Figure 4a), whereas an upsurge of inner dimension of end diastolic left ventricular (LVID) was noticed in CLP induced myocardial dysfunction group, which was effectively reduced by the administration of TG 50 and 100 mg/kg (Figure 4b).

### 2.5. Histopathology of Heart

**Figure 5:**
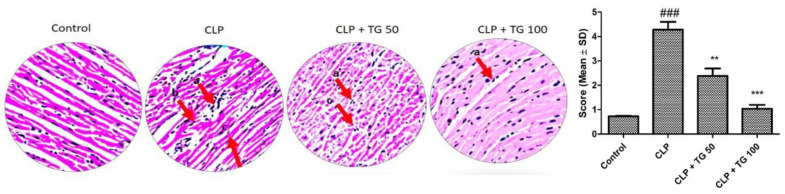
Effect of TG (50 and 100 mg/kg) on histological changes in the myocardial tissues at CLP-rats (hematoxylin-eosin, × 40, scale bar 5 mm= 25 µm). The pathologic are shown with arrows. Red arrows refer to CLP-induced areas in the myocardial tissues. a, myocardial cell necrosis; b, disrupted myocardial fiber; c, inflammatory cell infiltration. Values represented as means ± SD and n = 12/group. One-way analysis of variance (ANOVA), followed by Tukey’s test were used for statically analysis. Where ### *p* < 0.001 when compared to control group, whereas ** *p* < 0.01 and *** *p* < 0.001 when compared CLP group animals.

Hematoxylin and Eosin staining technique helped to see the structure of myocardiocytes. Control group heart histopathology showed no damage, degeneration, nor cell death A vibrant transverse strip of myocardial fiber was observed. The changes observed in diseased group and TG-treated groups were degeneration of cardiac muscle cell, separation of the center of cell, fractional breach of myocardial fiber, accumulation of fluid between the cells, and increasing the amount of red blood cells. All pathological changes in myocardial tissue of CLP group are reduced by the treatment of TG 50 and 100 mg/kg (Figure 5).

### 2.6. Estimation of Oxidative Markers

**Figure 6:**
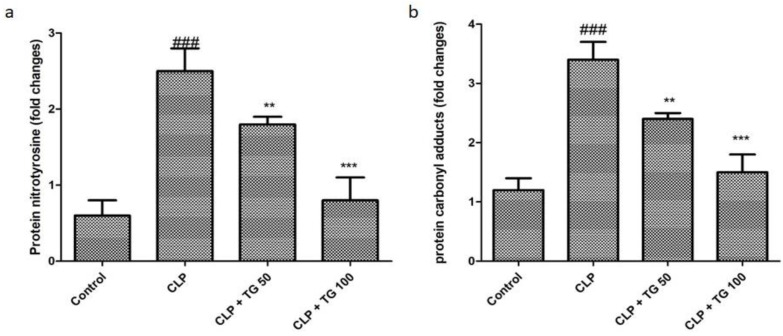
Cardiac oxidative markers: (**a**) protein nitration and (**b**) carbonyl content measured by quantitative ELISA (enzyme-linked immunosorbent assay). All markers were significantly increased in CLP-rats. TG (50 and 100 mg/kg) in significantly reduced CLP-induced oxidative stress markers, dose-dependently. Values represented as means ± SD and n = 12/group. One-way analysis of variance (ANOVA), followed by Tukey’s test were used for statically analysis. Where ### *p* < 0.001 when compared to control group, whereas ** *p* < 0.01 and *** *p* < 0.001 when compared CLP group of animals.

Quantitative determination of two different consecutive protein biomarker nitrotyrosine and carbonyl, which represent the level of oxidative stress, are illustrated by the ELISA (enzyme-linked immunosorbent assay) technique, and it shows profoundly increased 2.5 and 3.2, in succession. Increased amount of these two protein is caused by the CLP method, which was reduced in a dose-dependent manner by the treatment of TG 50 mg/kg and TG 100 mg/kg (Figure 6).

**Figure 7:**
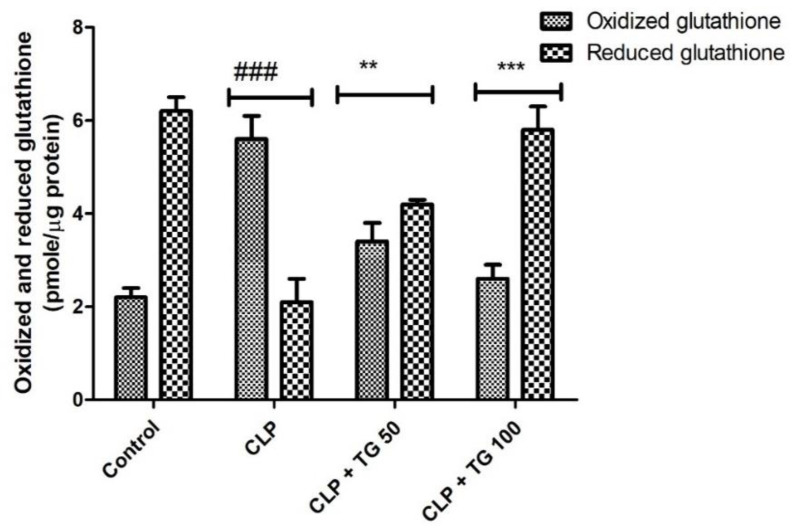
Treatment with TG (50 and 100 mg/kg) restores CLP-induced oxidized glutathione levels. CLP down regulated reduced glutathione, which was restored by TG (50 and 100 mg/kg), dose-dependently. Values represented as means ± SD; and n = 12/group. One-way analysis of variance (ANOVA), followed by Tukey’s test were used for statically analysis. Where ### *p* < 0.001 when compared to control group, whereas ** *p* < 0.01 and *** *p* < 0.001 when compared CLP group animals.

The level of antioxidant glutathione in oxidized and reduced form is also determined, and these levels of glutathione either oxidized form or reduced form are reversed by the treatment of TG 50 and 100 mg/kg in the dose-dependent manner (Figure 7).

### 2.7. Analysis of NOD-Like Receptor Family, Pyrin Domain Containing 3 (NLRP3) Expression

**Figure 8:**
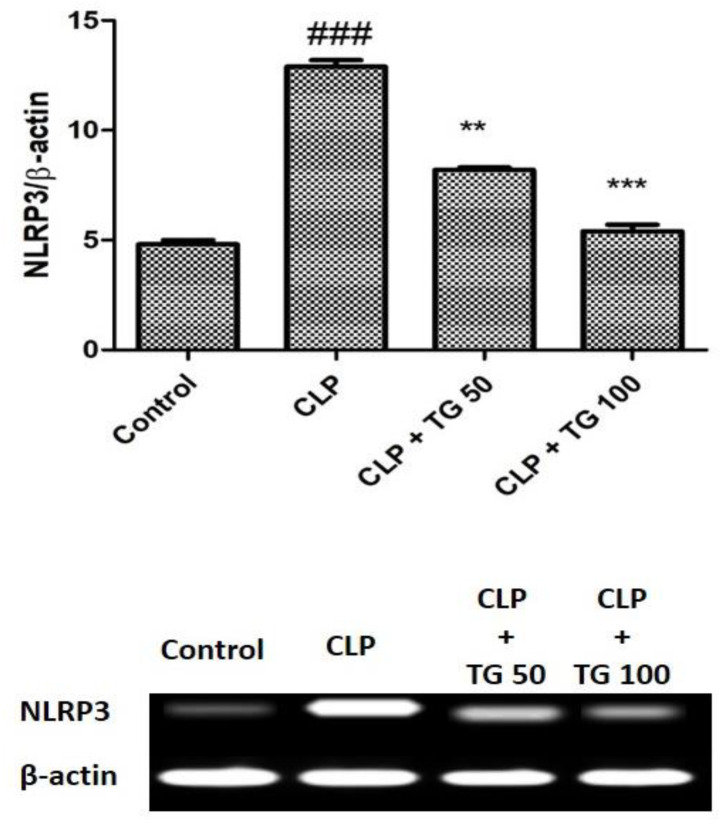
Relative nucleotide oligomerization domain (NOD)-like receptor family, pyrin domain containing 3 (NLRP3) expression. CLP increased the expression level of NLRP3 in myocardial tissue. TG (50 and 100 mg/kg) reduced the expression level of NLRP3 compared with CLP-treated rats at 24 h, dose-dependently. Values represented as means ± SD and n = 12/group. One-way analysis of variance (ANOVA), followed by Tukey’s test were used for statically analysis. Where ### *p* < 0.001 when compared to control group, whereas ** *p* < 0.01 and *** *p* < 0.001 when compared with CLP group animals.

We compared the septic and control group for the cellular expression of Nucleotide oligomerization domain (NOD)-like receptor family, pyrin domain containing 3 (NLRP3). It was significantly greater in septic rats as compared to control rat at the time of 24 h post-CLP surgery, while the cellular expression level of NLRP3 was decreased in septic rat by the treatment of TG 50 and 100 mg/kg in a dose-dependent manner, as expressed in Figure 8.

### 2.8. Analysis of Autophagy Markers Protein Light Chain 3 (LC)3, Cellular p62, PTEN/AKT/mTOR, and STAT3

**Figure 9:**
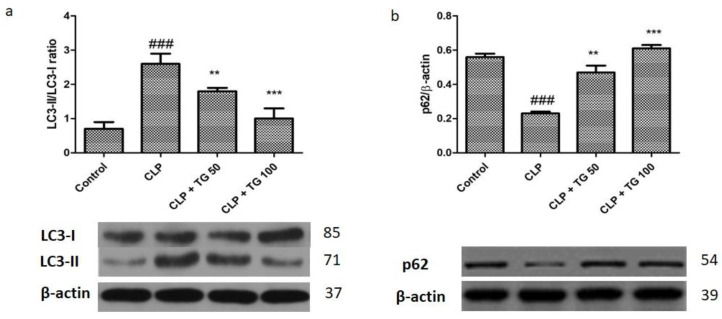
Treatment with TG (50 and 100 mg/kg) inhibits myocardial autophagy. Effect of TG (50 and 100 mg/kg) on autophagy markers LC3 (**a**) and p62 (**b**), along with control protein β-actin. Values represented as means ± SD and *n* = 12/group. One-way analysis of variance (ANOVA), followed by Tukey’s test were used for statically analysis. Where ### *p* < 0.001 compared to control group, whereas ** *p* < 0.01 and *** *p* < 0.001 compared CLP group animals.

Further, by using the Western blot technique we observed the changes in the cardiac protein p62 and LC3. During autophagy, cytoplasmic LC3 protein is lipidated and recruited to the autophagosomal membranes. The autophagosome fuses with the lysosome to form the autolysosome, and the breakdown of the autophagosome vesicle and its contents occurs. The ubiquitin-associated protein p62, which binds to LC3, is also a marker for autophagic flux. The fluctuation of these proteins indicated the autophagy in myocardial cells induced by sepsis. Modification in these proteins rapidly occurs, like increase in the rate of LC3-II/LC3-I, while reduction in the levels of p62 (*p* < 0.001) are seen in CLP induced sepsis (Figure 9a). The level of LC3-II/LC3-I significantly decreased, while the level of p62 (*p*< 0.05, *p*< 0.01 and *p*< 0.001) significantly increased in a dose-dependent manner after treatment with TG 50 and 100 mg/kg.

**Figure 10:**
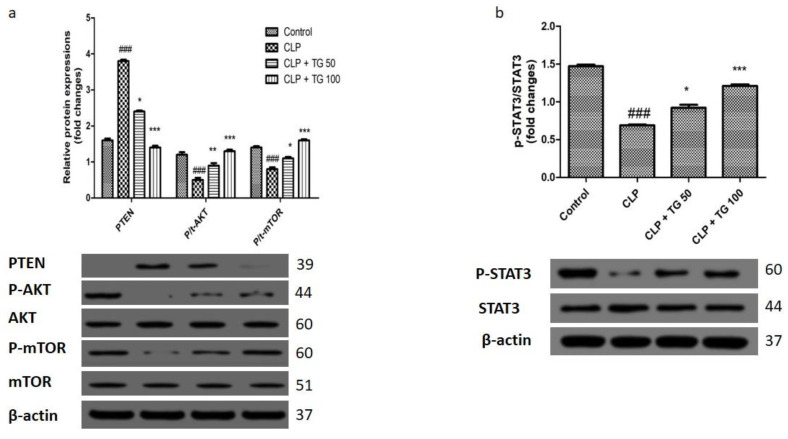
Effect of TG (50 and 100 mg/kg) on myocardial autophagy-related signal pathways and STAT3. (**a**) Treatment with TG (50 and 100 mg/kg) inhibits myocardial autophagy-related signal pathway PTEN/AKT/mTOR. (**b**) Treatment with TG (50 and 100 mg/kg) suppressed the expression of STAT3 in cardiac tissues. Values represented as means ± SDand *n* = 12/group. One-way analysis of variance (ANOVA), followed by Tukey’s test were used for statically analysis (Significance level * *p* < 0.05). Where ### *p* < 0.001 compared with control group, whereas ** *p* < 0.01 and *** *p* < 0.001 when compared with CLP group animals.

Earlier, it was reported that, for the regulation of autophagy, the phosphatidylinositol-3-kinase (PTEN/AKT/mTOR) is an essential signaling pathway, and it can be inhibited by the phosphatase and tensin homologue protein (PTEN). Western blot technique will help to examine and to evaluate the effect of TG 50 and 100 mg/kg on this signaling pathway by the measuring the level of the protein AKT, mTOR, and PTEN. The level of PTEN (*p* < 0.001) increases, and the expression of p-AKT (Ser473) and p-mTOR (Ser2448) (*p* < 0.001) decreases, by CLP. In a proper and significant dose of TG 50 mg/kg and TG 100 mg/kg treatment, the level of Phosphatase and tensin homolog reduced, while the level of p-AKT and p-mTOR (*p* < 0.05, *p* < 0.01 and *p* < 0.001) subsequently increases. By silencing the Phosphatase and tensin homolog protein in the cardiac cell, the signaling pathways (AKT/mTOR) were activated with the treatment of TG 50 mg/kg and TG 100 mg/kg, and this result suggested that inhibition of signaling pathway (PTEN-AKT/mTOR) by sepsis. There are seven members of the bi-functional protein of STAT family that undergo tyrosine phosphorylation. The Signal transducer and activator of transcription 3 (STAT3) in cytoplasm are involved in the signal transduction mechanism. Cellular expression of p-STAT3 (Y705) protein (*p* < 0.001) was significantly reduced by the action of CLP (Figure 10), while the level of p-STAT3 (*p* < 0.01 and *p* < 0.001) were increased with the treatment of TG 50 and 100 mg/kg in a dose-dependent manner.

## 3. Discussion

For the reprocessing of cytoplasmic content and removal of long-term protein present inside, the cells undergo autophagy which is an extremely regulated mechanism of bulk degradation. Homeostasis of cardiovascular tissue is maintained by the autophagy and is well known as cardiac remodeling process. It is also termed as a “double-edged sword”. In the current experimental paper, we examined the cardioprotective effect of two doses of TG, namely 50 and 100 mg/kg, in sepsis-induced myocardial damage developed by the surgical procedure of CLP. Firstly, there were marked reduction in injury indicative biomarkers and impairment of the myocardial cells after treatment with TG 50 and TG 100 mg/kg. Secondly, by inhibiting the autophagy, TG 50 and TG 100 mg/kg protected the cardiac cell from the cardiac injury, which was induced by sepsis. Thirdly, TG 50 and TG 100 mg/kg dose suppressed the PTEN to inhibit the autophagy in cardiac cells by activating the AKT/mTOR signaling mechanism [8,9].

Several factors contribute for the impairment of the cardiac function and endotoxin-mediated cardiac injury. Our experimentation showed improvement the levels of several biomarker, like Cardiac myosin light chain-1 (cMLC1), Cardiac troponin I (cTnl), lactate dehydrogenase, PARP activity, separation of deoxyribonucleic acid, and TUNEL staining a cell death marker. All these biomarkers were significantly decreased by the treatment of TG 50 mg/kg and 100 mg/kg. Cell death in the case of sepsis occurs by the both of the mechanism apoptosis and necrosis, different signaling pathway is also involved like overlapping pathway. Shrinkage of cell and loss of cytoskeleton are the major causes of impairments of cardiac function in apoptosis, whereas, predominantly in necrosis, cardiac function is diminished by the inflammatory responses. TG 50 mg/kg and TG 100 mg/kg showed dose-dependent reduction in the cell death and cardiac impairment manner [10,11].

The bioavailability and distribution of TG is higher and rapid in the systemic circulation after consumption in even small amount. In the experimental animal (rat) the pharmacokinetic data of TG shows presence in the systemic circulation for 10 days and by increasing the availability of TG, cardiac function may get improved. It is observed in the long-term study in rat that the left ventricular function of heart has been improved by the administration of TG in the case of diabetic cardiomyopathy. It also helps to improve the several heart disorders like ischemic heart disease and reperfusion of heart in the rat by using Langedorff assembly; thus, the data of TG obtained from current research will help to get rid of the cardiac disorder, and it will lead to the potential therapeutic benefit in the sepsis [12,13].

NOD-, LRR-, and pyrin domain-containing protein 3 are involved in the several diverse conditions like sepsis and auto inflammatory or infectious disease, it is single most important inflammasomes. It was assumed that pathological developments in septic injury are due to the unnecessary activation of NOD- LRR- and pyrin domain-containing protein 3 inflammasome. In the comparison of septic rat and control rat our finding are at 24 h post-CLP surgery the inflammasomes expression was significantly raised in septic rat, while TG in dose-dependent manner (50 and 100 mg/kg) will decrease the expression of inflammasomes in septic rat. This finding represents the reduction in inflammation of myocardial cells in septic rat by the dose-dependent treatment with TG 50 mg/kg and TG 100 mg/kg.

In earlier research it was shown that autophagic flux are the depiction of the whole autophagy which is a highly active process and by the development and evolution of autophagosomal via lysosomes fusion the autophagic flux were characterized. While the autophagy is always being controversial for their role in the heart because low level is beneficial while higher level leads to the damage of cardiac cells. So, it is always best to keep the moderate level of autophagy in septic condition which leads to reduce the cardiac injury or myocardial damage. It was believed that in the sepsis condition the autophagy will be initially activated due to the cell death it was followed by the ensuing phase of dysfunction. Yet, it is documented that against sepsis induced myocardial damage, blocking of autophagy activity is a cardioprotective in nature [14,15]. Our findings indicated that the autophagy was increased in the CLP rats. The CLP-induced autophagy was inhibited in cardiac tissue with the treatment of TG 50 mg/kg and TG 100 mg/kg. The modification in the LC3-II/I and p62 protein are indicative of increased autophagosomes.

A unique signaling pathway to regulate the autophagy in cardiac disease is PTEN/AKT/mTOR. The PTEN is a negative inhibitor of the signaling pathway. It was reported that in the glucose induced kidney disease or caused by the higher glucose level the oxidative stress suppressed by the mi-RNA 214 by the uncoupling protein and reactive oxygen species or Akt/mTOR pathway [15]. Through phosphatidylinositol 3-kinase-AKT signaling, TG can control the autophagy in the fibrosis of rat. It was a presupposition that PTEN is a primary target of TG, while it was reported by Hu et al. that, in colorectal cancer (CRC), by the blocking of ATG-12 facilitated autophagy, TG can lead the radio sensitivity. So, we have strong evidence that treatment of TG in a rat CLP rats regulated the PTEN/AKT/mTOR signaling pathway. The rise in the level of PTEN and reduced the p-AKT and p-mTOR level also indicate the inactivation of signaling pathway by the CLP. Besides, the data supported the theory that the level of PTEN reduced and level of p-AKT and p-mTOR will be increased by the treatment of TG, and, by suppressing the PTEN, TG will activate the signaling pathway and protective effect of TG against the sepsis-induced myocardial dysfunction [10,14].

In previous research, it was mentioned that STAT signaling pathway will be stimulated by blocking the activity of autophagy, which leads to the myeloid-derived suppressor cells accumulation and their immunosuppressive function. For the protection of heart and mitochondrial localization, the phosphorylation of STAT3 is necessary step [2,6]. Signaling pathway involved in the process can be modulated by the TG and our finding shows significant upsurge in the p-STAT3 by the treatment of TG 50 and TG 100 mg/kg. Activation of STAT3 by TG treatment can block the autophagy through the signaling pathway (PTEN/AKT/mTOR). With these findings, we propose a new therapeutic intervention for the management of sepsis induced myocardial injury.

## 4. Materials and Methods

### 4.1. Materials Used

TG was procured from Sigma-Aldrich, while β-actin, mTOR, p-mTOR, LC3-II/I, AKT, p-AKT, PTEN, STAT3, pSTAT3, and p62 antibodies were acquired from Cell Signaling Technology (Beverly, MA, USA). All the other chemicals and reagents used in the study were of analytical (AR) grade.

### 4.2. Animal Study

The Suresh Gyan Vihar University Committee on Ethical Use of Animals approved the standard operating procedures (SOPs) and experimental protocols related involving animal use and care. Suresh Gyan Vihar University’s animal laboratory provided 48 adult Sprague-Dawley male rats (weighing about 200–250 g). All the rats were acclimated for at least one week, under unique pathogen-free conditions, before the experiment following a 12-h day and night cycle. Animals were divided into 4 groups with *n* = 12 in each group as normal group, CLP Group, CLP + TG Group (50 mg/kg), CLP + TG Group (100 mg/kg). Briefly, CLP sepsis model was developed in three groups, the disease control and the diseased with treatment at 2 dose levels 50 mg/kg and 100 mg/kg, as follows. Rats were anaesthetized in a small animal ventilator with inhalation of isoflurane (anesthesia induced by 3% and sustained by 0.5%). Cecum was exposed and ligated at two points. Using 18-gauge needle, the exposed cecum was punctured at 2 places. Feces were gently extruded, and cecum was placed back in its anatomical position [16,17].

TG were administered orally by dissolving in water. Heart and blood samples were collected after rapidly anaesthetizing via chloralic hydras at the end of experiment [12].

### 4.3. Tissue and Cardiac Injury Markers Analysis

Using an automated analyser (Thermo Scientific, Vantaa, Finland), CK and LDH levels of plasma were calculated. ELISA-based assaywere used for the determination of concentration of plasma cTnI and PARP as per the manufacturer’s (Life Diagnostics Inc., West Chester, PA, USA) protocol, were used for plasma cMLC1 [10].

### 4.4. Echocardiography

Echocardiographic cardiac parameters were measured are conducted as previously described [11].

### 4.5. Histological Analysis

Euthanasia was done 24 h post-CLP with rats and paraformaldehyde was used for fixing the heart. Hematoxylin and eosin stains were used for the staining purpose after the rat’s heart was being dehydrated, seeded, dipped in wax, embedded and sectioned. The light microscopy (Olympus, Tokyo, Japan) was used for the observation of tissue changes.

### 4.6. Cardiac TUNEL Staining

In line with the earlier published instructions provided by manufacturer’s, In Situ Cell Death Detection Kit (Roche, Mannheim, Germany) were used, and cardiac TUNEL staining was done [18].

### 4.7. Cardiac Cell Death Markers

ELISA-based kit (Roche) was used for measurement of DNA fragmentation and HT Universal Colorimetric PARP Assay Kit (Trevigen, Gaithersburg, MD, USA) was used for the PARP activity [19].

### 4.8. RT-PCR for NLRP3 Expression

A one-step RT-PCR kit (GeneCopoeia, Rockville, MD, USA) was used for conduction of trizol method and reverse transcribed to extract total RNAs from cardiac tissue. The primer sequences which are used are: NLRP3 forward, 5′-CTGTTCTCATGGGTTGGGGC-3′ and reverse, 5′-GACTCCTGAGTCTCCCAAGGC-3′.

### 4.9. Cardiac Glutathione Level

GSR-DTNB recycling assay was used for the determination of cardiac glutathione levels from tissue lysates [20].

### 4.10. Cardiac Oxidative Stress Markers

OxiSelect Nitrotyrosine ELISA Kit was used for the determination of Protein nitrotyrosine nitration and Protein Carbonyl Colorimetric Assay Kit was used for the determination of the carbonyl content in tissue lysate protein [21].

### 4.11. Western Blot

Standard protocols were used for performing the Western blot analysis and the hearts of the mice were used for the preparation of total proteins. 250 mM sucrose buffer was used for the isolation of total protein extracts from the heart of rats, and Cell Signaling Technology (Beverly, MA, USA) was the source of purchase of primary antibodies against β-actin, mTOR, p-mTOR, LC3-II/I, AKT, p-AKT, PTEN, STAT3, pSTAT3, and p62. NIH Image software (Bethesda, MD, USA) was used for densitometric analysis, and scanning of band images were done.

### 4.12. Statistical Analysis

The Prism 8.0.2 software (GraphPad Software Inc., San Diego, CA, USA) was used for the statistical analysis of the data. All the data were expressed as mean ± standard error of the mean (SEM). The data was first tested for normality and homogeneity and analyzed using a one-way analysis of variance (ANOVA), followed by Tukey’s test given the 5% significance level [22].

## 5. Conclusions

By modulating PTEN/AKT/mTOR signaling pathway and inhibiting the autophagy, TG reduced the sepsis induced myocardial tissue damage. The findings of the present mechanistic investigation justify the therapeutic effect of TG on myocardial damage. However, more clinical data on safety and dose optimization studies are required for successful translation of TG as a cardioprotective drug.
